# Electrospun Polycaprolactone (PCL) Degradation: An In Vitro and In Vivo Study

**DOI:** 10.3390/polym14163397

**Published:** 2022-08-19

**Authors:** Juliana R. Dias, Aureliana Sousa, Ana Augusto, Paulo J. Bártolo, Pedro L. Granja

**Affiliations:** 1 Center for Rapid and Sustainable Product Development (CDRsp), Polytechnic Institute of Leiria, 2030-028 Marinha Grande, Portugal; 2 Instituto de Investigação e Inovação em Saúde (i3S), Universidade do Porto, 4200-135 Porto, Portugal; 3 Instituto de Engenharia Biomédica (INEB), Universidade do Porto, 4200-135 Porto, Portugal; 4 MARE-Marine and Environmental Sciences Center, ARNET, ESTM, Instituto Politécnico de Leiria, 2520-630 Peniche, Portugal; 5 Singapore Center for 3D Printing, Nanyang Technological University, 22 Jurong West, Singapore 639798, Singapore

**Keywords:** polycaprolactone, electrospinning nanofibers, degradation, enzymatic, hydrolytic, in vitro, in vivo

## Abstract

Polycaprolactone (PCL) is widely used in tissue engineering due to its interesting properties, namely biocompatibility, biodegradability, elastic nature, availability, cost efficacy, and the approval of health authorities such as the American Food and Drug Administration (FDA). The PCL degradation rate is not the most adequate for specific applications such as skin regeneration due to the hydrophobic nature of bulk PCL. However, PCL electrospun fiber meshes, due to their low diameters resulting in high surface area, are expected to exhibit a fast degradation rate. In this work, in vitro and in vivo degradation studies were performed over 90 days to evaluate the potential of electrospun PCL as a wound dressing. Enzymatic and hydrolytic degradation studies in vitro, performed in a static medium, demonstrated the influence of lipase, which promoted a rate of degradation of 97% for PCL meshes. In an in vivo scenario, the degradation was slower, although the samples were not rejected, and were well-integrated in the surrounding tissues inside the subcutaneous pockets specifically created.

## 1. Introduction

When skin damage occurs, the standard procedure relies in applying a wound dressing due their efficiency, low cost, and availability [1]. The dressing has, as its main functions, to promote a moist environment in the wound and to protect the wound against mechanical injury and microbial contamination, especially during the inflammatory stage [2].

Electrospun wound dressings present exceptional properties compared to conventional dressings such as promotion of the hemostasis phase, enhanced wound exudate absorption, semi-permeability, easy conformability to the wound, functional ability, and the possibility of not inducing scar formation [3]. Ideally, the dressing should be able to fit to the wound shape, absorb wound fluid without increasing the bacterial proliferation or causing excessive dehydration, provide pressure for hemostasis, and prevent leakage from occurring [4]. The dressing should also support the wound and surrounding tissues, eliminate pain, promote re-epithelialization during the reparative phase, be easily applied and also be removed with minimal injury to the wound [5]. According to tissue engineering (TE) principles, it is expected that a tissue engineered device is able to promote tissue regeneration and, at the same time, degrade, ideally at the same rate [6]. Based on this principle, the materials used, and their degradation rates are a relevant concern. One of the most widely used synthetic polymer in TE is polycaprolactone (PCL) due to its biocompatibility, structural stability, and good mechanical properties coupled with biodegradability [7,8]. However, as a result of the semi-crystalline and hydrophobic nature of PCL, it presents a slow degradation rate (2–4 years) that limits its application as a wound dressing for skin regeneration [6]. The degradation rates of PCL electrospun fibers showed some changes compared to the bulk PCL as a consequence of a larger surface area-to-volume ratio and reduced crystallinity, which result from the electrospinning process [9].

In this work, an evaluation of the degradation kinetics of PCL electrospun meshes in vitro and in vivo were performed. For the first time, the comparison of hydrolytic and enzymatic in vitro degradation with in vivo assays was carried out. PCL is a biodegradable aliphatic polyester and, as a consequence, the enzyme lipase that hydrolyzes the ester bonds was selected [9,10]. There are different sources of lipase in the human body such as leukocytes, present in the wound healing process, with the lipase concentration of healthy adults in the range of 30–190 U/L [11,12]. Thus, the in vitro degradation of the PCL meshes was monitored both in phosphate buffered saline (PBS) alone or enzymatic media. The degradation kinetics were evaluated through the quantification of weight lost, swelling degree, thermal behavior, molecular weight changes, and mechanical properties. In vivo studies were performed through the implantation of PCL electrospun meshes in subcutaneous pockets. After the pre-clinical assays, degradation was assessed by histology and histochemistry.

## 2. Materials and Methods

### 2.1. Electrospun Mesh Preparation

Poly(ɛ-caprolactone) (PCL) was supplied by Perstorp (Mw: 50,000 g·mol^−1^; bulk density: 1.1 g·cm^−3^) and dissolved in acetone (dimethyl ketone or DMK) from Sigma-Aldrich (St. Louis, MO, USA). A PCL/acetone solution (17 wt-%) was prepared by dissolving the polymer and stirring it at 37 °C overnight. The samples were produced using homemade electrospinning equipment at room temperature (RT), with a constant flow rate of 3.17 mL·h^−1^ (syringe pump model SP11Elite; Harvard Apparatus, Holliston, MA, USA), 10 cm distance between the syringe and collector, and 10 kV voltage.

### 2.2. In Vitro Degradation Profile

The samples were prepared as rectangles with 10 × 40 mm dimensions, an average thickness of 166 ± 40 µm, and average weight of 10.16 ± 2.52 µg. Two different sets were prepared: for the first set, each specimen was immersed into a capped bottle containing 10 mL of phosphate-buffered saline (PBS; 8.00 g NaCl, 0.20 g KCl, 1.44 g Na_2_HPO_4_·12H_2_O, 0.20 g KH_2_PO_4_ in 1 L distilled H_2_O, pH 7.4) and 0.02% (*w*/*v*) of sodium azide from Sigma-Aldrich (St. Louis, MO, USA) as a bacteriostatic agent; for the second set, each specimen was immersed into a capped bottle containing 10 mL PBS with 150 U·L^−1^ of lipase concentration (amano lipase from Burkholderia cepacia; Sigma-Aldrich (St. Louis, MO, USA)) and 0.02% (*w*/*v*) of sodium azide. The bottles were incubated at 37 °C with constant shaking of 100 rpm for 90 days. Fifteen samples were tested for each condition (PBS or PBS/lipase) with the exception of samples in PBS/lipase after 28 days, when only six samples per time-point were tested due to their almost total degradation. All PBS solutions were changed weekly, and PBS/lipase was chanced twice a week to assure that enzymatic activity was maintained.

### 2.3. Morphology, Apparent Density, Porosity, and Surface Area

The morphology of the electrospun meshes was examined by scanning electron microscopy (SEM) using a Quanta 400 FEG ESEM/EDAX Genesis X4M (FEI, Hillsboro, OR, USA). Prior to examination, samples were coated with an Au/Pd thin film by sputtering (Sputter Coater, SPI, West Chester, PA, USA). SEM images were also used to evaluate the fiber diameter distribution using ImageJ software (Fiji, version J1.46r., NIH, Bethesda, MD, USA). For each condition, three individual samples were analyzed and 50 measurements per image were performed. The apparent density and porosity of the electrospun meshes were calculated using Equations (1) and (2) [13], respectively, where the thickness of the meshes was measured with a micrometer.

(1) $$\mathrm{Apparent}\;\mathrm{density}\;\left(g{\cdot \mathrm{cm}}^{-3}\right)=\frac{\mathrm{Mesh}\;\mathrm{mass}\;(g)}{\mathrm{Mesh}\;\mathrm{thickness}\;\left(\mathrm{cm}\right){\;\times \;\mathrm{Mesh}\;\mathrm{area}\;(\mathrm{cm}}^{2})}$$

(2) $$\mathrm{Mesh}\;\mathrm{porosity}=\left(1-\;\frac{\mathrm{Mesh}\;\mathrm{apparent}\;\mathrm{density}\;\left(g{\cdot \mathrm{cm}}^{-3}\right)}{\mathrm{Bulk}\;\mathrm{density}\;\mathrm{of}\;\mathrm{PCL}\;\left(g{\cdot \mathrm{cm}}^{-3}\right)}\right)\;\times \;100$$

The surface area to volume ratio (SA/v) for the electrospun PCL meshes was also calculated.

### 2.4. Weight Loss and Water Uptake

At each time-point (7, 14, 28, 42, 63, 77, and 90 days), the samples were removed from the buffer solution, washed with distilled water, and the excess water was removed using filter paper. Following this, the samples were weighed to evaluate the swelling degree, as shown in Equation (3). Next, the samples were incubated for 24 h at 37 °C and the weight loss was quantified according to Equation (4).

(3) $$\mathrm{Swelling}\;\mathrm{degree}\;\left(\%\right)=\frac{W_{w}{-W}_{d}}{W_{0}}\;\times \;100$$

where W_W_ is the wet weight; W_d_ is the dry weight after degradation; and W_0_ is the initial dry weight.

(4) $$\mathrm{Weight}\;\mathrm{loss}\;\left(\%\right)=\frac{W_{0}{-W}_{d}}{W_{0}}\;\times \;100$$

where W_0_ is the initial weight and W_d_ is the dry weight, after the degradation tests.

### 2.5. Thermal Analysis

The thermal properties and stability were determined using simultaneous thermal analysis (STA), recurring to a 6000 system (Perkin Elmer, San Jose, CA, USA). Samples of 1.5–2.5 mg were placed into ceramic pans and the tests were performed under dry nitrogen purge (flow rate of 20 mL·min^−1^). Samples were submitted to heating from 30 to 450 °C at 10 °C·min^−1^. Melting temperatures (T_m_) were obtained at the peak of the melting endotherms. The enthalpies of fusion ($\Delta H_{m}$) were obtained from the areas under the peaks. The In and Ag samples were used as the calibration standards. The crystallinity degree ($X_{c}$) was determined according to Equation (5) [14].

(5) $$X_{c}\;\left(\%\right)=\frac{\Delta H_{m}}{w\;\times \;\Delta H_{m}^{0}}\;\times \;100\;$$

where $\Delta H_{m}$ is the experimental melting enthalpy and w is the weight fraction of material. Additionally, for $\Delta H_{m}^{0}$, which corresponds to the enthalpy of melting of 100% crystalline PCL, the value from the literature of 139 J·g^−1^ was assumed [15,16].

### 2.6. Determination of Molecular Weight

The molecular weight (M_w_) distribution of the samples was determined using gel permeation chromatography/size exclusion chromatography (GPC/SEC) composed of a pump and autosampler (GPC Max, Viscotek, Malvern Panalytic, Workcester, UK), a dual detector (model T60A, Viscotek, Malvern, Workcester, UK), and a refractive index detector (model K2301, Knauer, Berlin, DE, Germany). The GPC/SEC was equipped with PLgel mixed-B columns (Agilent Technologies, Santa Clara, CA, USA) of 7.5 × 300 mm and 10 µm porosity, composed of a polystyrene dinivylbenzene copolymer. Samples were individually dissolved in tetrahydrofuran (THF; 99.7% purity) (HiperSolv Chromanorm, VWR, Radnor, PA, USA). Then, 100 µL of the solution was injected into the GPC, which had been previously calibrated with poly(methlylmethacrylate) (PMMA; Polycal, Malvern Panalytic, Workcester, UK) standards in THF with known molecular weights of 64,898 and 95,081 Da. Distilled THF with a flow rate of 1 mL·min^−1^ was used as the mobile phase. For each scaffold type, three samples were collected, each of which was analyzed in triplicate. The average molecular mass distributions were determined using Omnisec Viscotek software (Malvern Panalytic., Workcester, UK), version 4.6.2.359.

### 2.7. Determination of Wettability through Contact Angle Measurement

The static contact angle was determined by the sessile drop method by the use of an optical tensiometer (model Theta Lite; Biolin Scientific, Manchester UK), through the measurement of the angle formed between a water droplet and the PCL mesh surface. The 20 μL deionized water was slowly titrated on the surface of the electrospun membrane and the morphology of the water droplets was recorded.

### 2.8. Mechanical Testing

The elongation at break and Young’s modulus of the PCL electrospun meshes were determined using a texturometer (model TA. XT Plus; Stable Micro System, Manchester, UK) with a 5 N load-cell. Testing was carried out in a controlled environment at RT and relative air humidity of 45%. The gauge length was 15 mm, and the test speed was 1 mm s^−1^. At least five individual samples were tested from each sampling group.

### 2.9. Subcutaneous Implantation

The electrospun meshes were prepared in a circular shape with a 0.8 mm diameter and sterilized with UV light prior to implantation. All animal experiments were conducted following protocols approved by the Ethics Committee of the Portuguese authority on the animal welfare and experimentation (DGAV) and were performed at the Faculty of Medicine of the University of Porto. CD1 male mice were housed at 22 °C with a 12 h light/dark cycle and had ad libitum access to water and food. For each experimental group (7, 60, and 90 days), six animals were used and four electrospun meshes were subcutaneously implanted in the back of the subcutaneous pockets created in each animal. The animals were anesthetized with ketamine/xylazine (Sigma Aldrich, St. Louis, MO, USA) (0.1 mL/20 g mouse at final dosage of 87.5 mg kg^−1^ ketamine/12.5 mg kg^−1^ xylazine) and anesthesia was maintained over the course of surgery by continuous isoflurane (Sigma Aldrich, St. Louis, MO, USA) delivery. The dorsal surgical sites were shaved and sterilized. Four subcutaneous pockets were created per mouse for the insertion of the PCL electrospun membranes. After implantation, incisions were closed with sutures and analgesics were administrated (tramadol (Sigma Aldrich, St. Louis, MO, USA)). The animals were routinely monitored for their general appearance, activity, and healing of the implant sites, and were euthanized after 7, 60, and 90 days for implant retrieval. No animals were lost during the study.

### 2.10. Histology by Hematoxylin and Eosin (H&E) Staining

For histological evaluation, the implantation sites were harvested, which included the entire PCL electrospun mesh and some surrounding tissue and fixed in 4% (*v*/*v*) paraformaldehyde (Sigma Aldrich, St. Louis, MO, USA) overnight. Samples were dried through a series of graded alcohol baths and in xylene (Sigma Aldrich, St. Louis, MO, USA), embedded in paraffin (Sigma Aldrich, St. Louis, MO, USA) and sectioned in 5 µm thick slices. Sections were stained with H&E (Beyotime, Shanghai, China).

### 2.11. Statistical Analysis

All data points were expressed as the mean ± standard deviation (SD). Statistical analyses (Levene’s and *t*-test) were carried out using SPSS Statistics 20. software 0 (IBM, Armonk, NY, USA) with a 95% confidence level. The results were considered statistically significant when *p* ≤ 0.05 (*).

## 3. Results

### 3.1. Physical Characteristics after In Vitro Degradation Studies

As previously described, the in vitro degradation of the electrospun PCL meshes was monitored in different media (PBS and PBS/lipase) and characterized by several distinct techniques.

**Figure 1:**
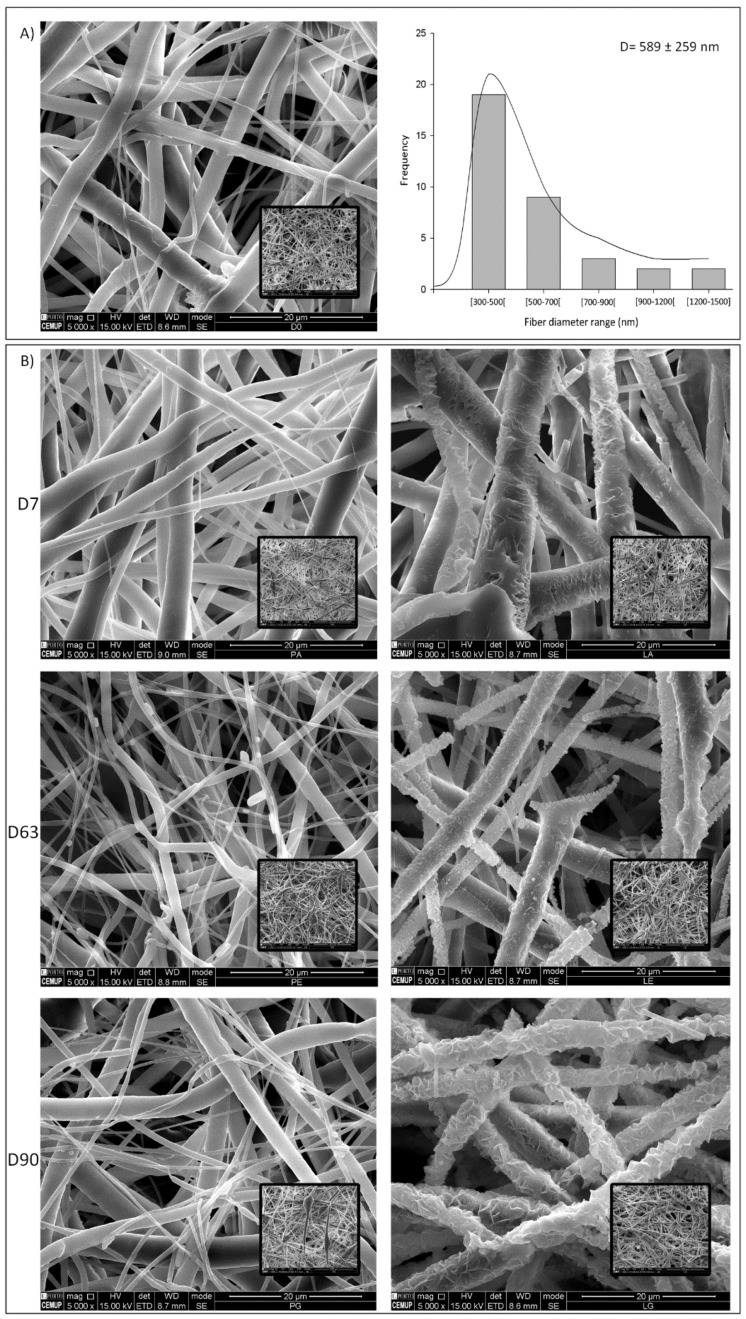
The SEM representative images. (**A**) Left: electrospun meshes morphology before degradation; right: fiber diameter distribution; (**B**) PCL electrospun meshes degradation for different periods of time (7, 63, and 90 days); left: hydrolytic degradation; right: enzymatic degradation. White bars correspond to 20 µm and insets represent amplifications of 1000 times.

**Figure 2:**
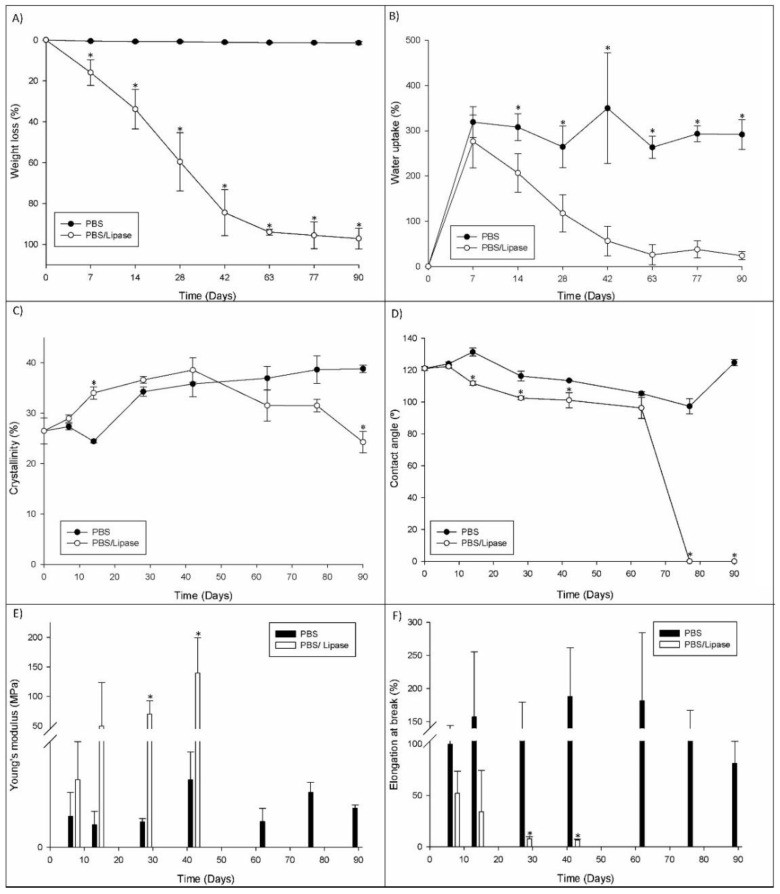
The results of the PCL electrospun meshes’ degradation in PBS and PBS/lipase medium over time. (**A**) Weight loss; (**B**) Water uptake; (**C**) Crystallinity; (**D**) contact angle; (**E**) Young’s modulus; (**F**) elongation at break. * Statistically significant difference (*p* ≤ 0.05) compared to the homologous time-point of enzymatic degradation.

SEM images (Figure 1) show the morphology of non-degraded and degraded electrospun meshes, according to different periods of time in media with or without the enzyme. As shown in Figure 1B (right), enzymatic degradation occurred at the material surface, increasing the surface roughness of the meshes with time and consequently, a massive decrease in the overall fiber diameter. For the TE applications, it is appropriate that the fibers undergo gradual degradation, leaving enough space for new tissue ingrowth. Comparing Figure 1B (right) with Figure 2A, it was noticeable that the presence of the enzyme accelerated the degradation process, as expected. It is worth mentioning that the rougher shape of the meshes after degradation in enzymatic media (Figure 1B right) and the longer the period of time, the more pronounced they became (maximum at 90 days).

The scaffolds must constitute a highly porous and fully interconnected network to provide a surface area large enough to allow for cell ingrowth, uniform cell distribution, and facilitate the neovascularization of the structure [17,18,19]. An ideal scaffold needs to have an open and interconnected porous network and a high degree of porosity (>60–90%) to interact and integrate with the host tissue [20]. According to Equations (1) and (2), the obtained value for the porosity of the electrospun meshes produced was 86.06%.

For the most part, cells used in TE are anchorage-dependent, and therefore, the scaffold should facilitate their attachment. Hence, scaffolds with a large and easily accessible surface area are more adequate in order to hold the number of cells required to replace or restore tissue or organ functions [21]. The approximate ratios of the electrospun meshes were determined (Table 1) and the results demonstrated that the produced electrospun meshes resulted in an increasingly higher surface area/volume ratio (SA/v = 14.3 mm^−1^) when compared to other structures from the same material such as a solvent cast film of PCL, which was 10.26 mm^−1^ [6].

The weight loss is a direct measurement to quantify polymer degradation. When the long polymer chains are cleaved to shorter ones, the weight of the original fiber is reduced [22]. According to the weight loss results, the enzymatic degradation resulted in a massive sample degradation (Figure 2A). In the PBS medium, the weight loss was not significant, reaching only 1.44% after 90 days. On the other hand, the degradation in the PBS/lipase medium showed fast degradation until day 42, reaching 84.42% of weight loss. After that, the degradation became slower, reaching 97.11% of weight loss after 90 days. However, with the remaining fragments obtained after 90 days of degradation, it was possible to acquire some SEM images that, despite presenting a huge fiber erosion, cannot be representative of the massive degradation achieved, since the fragments that are not fully degraded are the result of the low permeability of that sample area.

Upon implantation, biomaterials interact with the surrounding fluids, initially by uptaking them, thus promoting the degradation process [23]. The water uptake makes the materials more flexible and promotes changes in the dimensions of the implant material. Simultaneously, a higher water uptake enhances the hydrolysis process [23]. In Figure 2B, it is possible to observe the water uptake in both media, showing that their profile was not constant, which could be due to the continuous degradation of samples not allowing for the evaluation with constant weight along time. The swelling degree profile showed that, despite the PCL having a hydrophobic character, the meshes with high porosity exhibited a great capability to retain water. The average values for the PBS media were above 200% of the water uptake, over all degradation periods. However, in the PBS/lipase medium, due to the massive degradation observed, the water uptake decreased over time. Moreover, in enzymatic degradation, fiber erosion occurred at their surface, demonstrating that the degradation mechanism is not dependent on the material hydrophilicity [16].

STA was used to assess the thermal behavior of the degraded PCL electrospun meshes. The results obtained corresponding to the thermogravimetric (TG) effects coupled with the recording of a differential scanning calorimetry (DSC) heat flow signal. The TG and DTA/DSC analysis methods were applied simultaneously to the non-processed and processed PCL and PCL electrospun meshes degraded in different media. The values of melting temperature (T_m_) and degradation temperature (T_deg_) are presented in Table 2, and the PCL crystallinity degree for all samples is presented in Figure 2C. The PCL grain results showed a T_m_ = 63.2 ± 0.4 °C and a crystallinity degree of 351.6 ± 2.3%. On the other hand, the PCL processed by electrospinning presented slightly lower values compared to the raw material, namely concerning T_m_ (62.7 ± 0.7 °C) and crystallinity (26.49 ± 2.6%). Several authors have correlated the fast solvent evaporation with the low molecular arrangement in the polymer, leading to a crystallinity decrease in the electrospun fibers [24,25]. The Tm is determined by the energy state of the sample, which, in turn, is often affected by the processing. The reduction in Tm in the electrospun samples compared to the raw material might be due to the polymer chain orientations that change during the electrospinning process [26]. The meshes’ degradation in PBS/lipase presented a turning point at day 42, at which point a rise in the crystallinity could be observed, coupled to an increase in Tm due to the rapid degradation of the amorphous part, as demonstrated by the weight loss. After that, the weight loss rate decreased as well as the crystallinity degree, which can be correlated with the degradation of the polymer crystalline part [24]. The samples degraded in PBS showed a slight weight loss that corresponded only to the degradation of the amorphous part, thereby resulting in the crystallinity increase as the Tm increased [25].

During the degradation period, the Mw of the PCL electrospun meshes were measured. In spite of the PCL Mw indicated by the supplier of 50 kDa, the GPC results revealed a slightly lower value of 45.5 ± 6.6 kDa. Degradation can occur through two different mechanisms: surface erosion or bulk degradation. Consequently, the changes in Mw and weight loss during the degradation period allows for differentiation between the two mechanisms [27]. Hydrolytic degradation is characterized by bulk degradation and, for that reason, despite the slight weight loss, a Mw decrease was observed. On the other hand, the enzymatic degradation reached 97.1% of weight loss, although the Mw remained close to the initial value, probably due to a surface erosion mechanism. In terms of the polydispersity index (PDI), which allows for the evaluation of the samples’ heterogeneity based on size, it was possible observe that all samples showed values above 1, meaning that all samples presented a broad size distribution [28].

The water uptake of biodegradable polymers indicates its hydrophilic/hydrophobic characteristic and, consequently, their susceptibility to degradation through hydrolytic processes [29]. To evaluate the hydrophilic character of the electrospun meshes, the contact angle between the meshes and water droplets was measured. As is well-known, the PCL has a hydrophobic character and the nanofibrous meshes produced through electrospinning exhibited an angle of 121.09 ± 1.18° (Figure 2D), meaning that the electrospun meshes also exhibited a hydrophobic behavior. 

To evaluate the mechanical properties of the degraded electrospun meshes, stress–strain tests were performed. For each degradation time, five consecutive tensile tests were measured for five independent samples. The values for the Young’s modulus and the elongation at break obtained can be observed in Figure 2E,F. According to the literature, several studies have been performed to evaluate the Young’s modulus and elongation at the break of skin through tensile tests, with the average values presenting very large intervals, namely E = 2.9–150.0 MPa and ƐB = 17–207% [30,31,32,33]. The results obtained for the electrospun sample meshes without degradation, namely E = 8.16 ± 3.56 MPa and ƐB = 221.2 ± 65.8%, were in those intervals [30,31,32,33]. The samples degraded in the enzymatic medium showed a progressive increase in the Young’s modulus until day 28, after which it suffered a slight decrease until day 42, in agreement with what has been reported elsewhere [16,34]. This trend was correlated with the increase in crystallinity (as seen on thermal analysis results), showing that increasing the crystallinity also caused an increase in the Young’s modulus (along with the decrease of elasticity) as a result of the sharp degradation. Concerning the elongation at break, a decrease with the time of degradation was observed, probably as a result of the high degradation rate that turned the samples less elastic and more brittle. The E and Ɛ values of the samples in the PBS medium showed a non-consistent trend during the whole degradation period, which could result from only a slight degradation.

### 3.2. Behavior after In Vivo Degradation

Implantation studies were carried out to assess the in vivo degradation of the PCL electrospun meshes produced. After the implantation and retrieval of the electrospun meshes, the specimens were routinely processed for histology, and transversal sections were analyzed by standard H&E staining.

**Figure 3:**
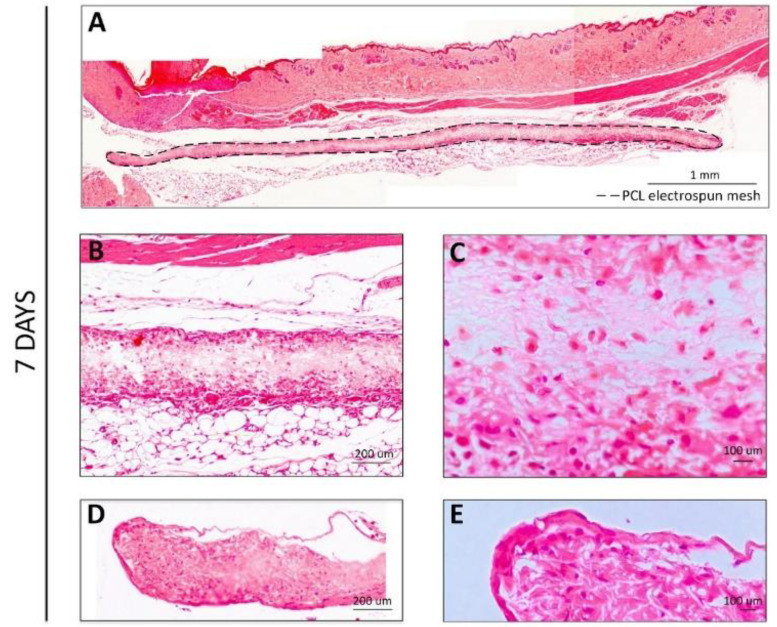
H&E staining for the PCL electrospun meshes implanted after 7 days. (**A**) Reconstruction of the full membrane; (**B**,**C**) Interior membrane sections; (**D**,**E**) End-limits of the meshes.

After 7 days of subcutaneous implantation (Figure 3), the meshes presented a macroscopic aspect similar to the hydrated meshes prior to implantation. Few adhesion sites were observed, and no major alterations were detected. Image reconstruction of the full membrane (Figure 3A) showed that the membranes remained parallel to the skin of the animal below the muscular layer. H&E staining images at higher magnifications revealed that a low foreign body response was observed at the surface of the membranes (Figure 3B), possibly caused by multiple reactions of various inflammatory cells (neutrophils, eosinophils, basophils, and others) [35]. In the interior part of the mesh, some cells could already be observed, probably granulocytes, namely polymorphonuclear (PMN) and eosinophils (Figure 3B,C, dark pink), although the majority of the area was still occupied by the PCL nanofibers (Figure 3C, light pink) [35,36]. The same could be observed throughout the mesh, even when the exposed mesh area was higher, at the end-limits of the meshes (Figure 3D,E).

**Figure 4:**
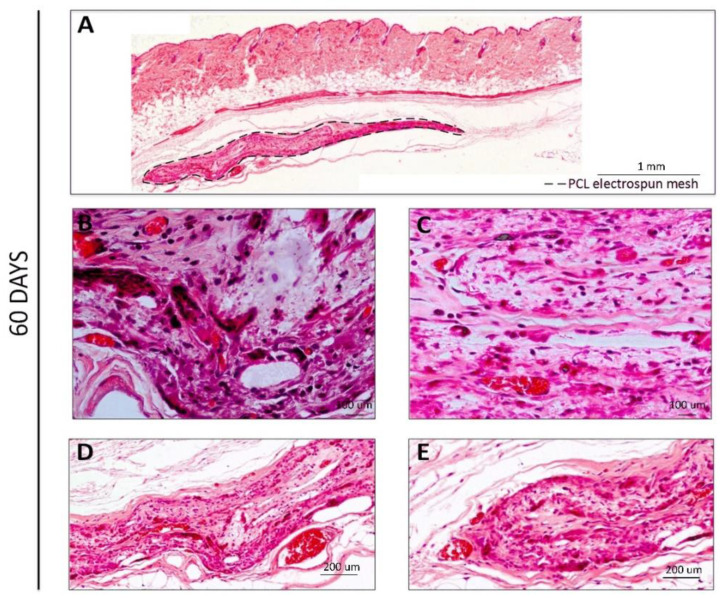
H&E staining for the PCL electrospun meshes implanted after 60 days. (**A**) Reconstruction of the full membrane; (**B**,**C**) Interior membrane sections; (**D**,**E**) End-limits of the meshes.

**Figure 5:**
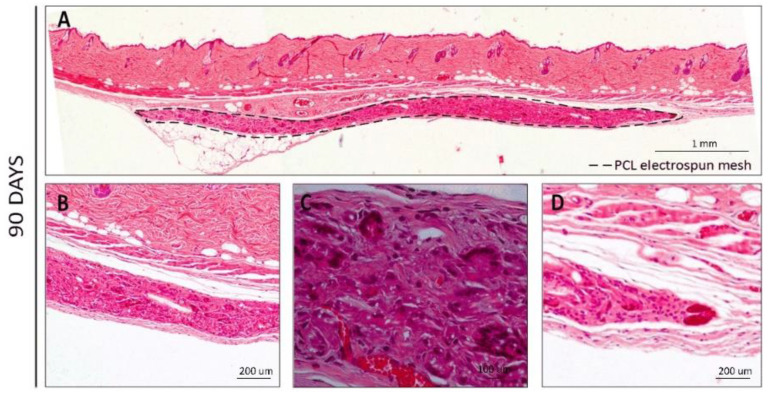
H&E staining for the PCL electrospun meshes implanted after 90 days. (**A**) Reconstruction of the full membrane; (**B**,**C**) Interior membrane sections; (**D**) End-limit of the mesh.

At later time points (60 and 90 days, Figure 4 and Figure 5, respectively), a higher cell infiltration was clearly present through the interior/non-exposed area of the meshes. After 60 days of implantation, the retrieval was harder to achieve since the macroscopic observation of the meshes revealed translucent meshes, but still presenting the circular format. When analyzing histological sections (Figure 4), the increased cell number in the interior of the meshes could be clearly observed and blood vessels could also be distinguishable within the mesh (Figure 4B–E, with erythrocytes in bright red), demonstrating that the electrospun PCL mesh was clearly being integrated in the host tissue. Furthermore, hollow spaces could also be observed, indicating that the material was being degraded.

A very similar pattern could be observed on the H&E sections at 90 days post-implantation (Figure 5), where larger blood vessels containing red blood cells could be identified and a stronger collagen staining (pink) was present (Figure 5B–D).

**Figure 6:**
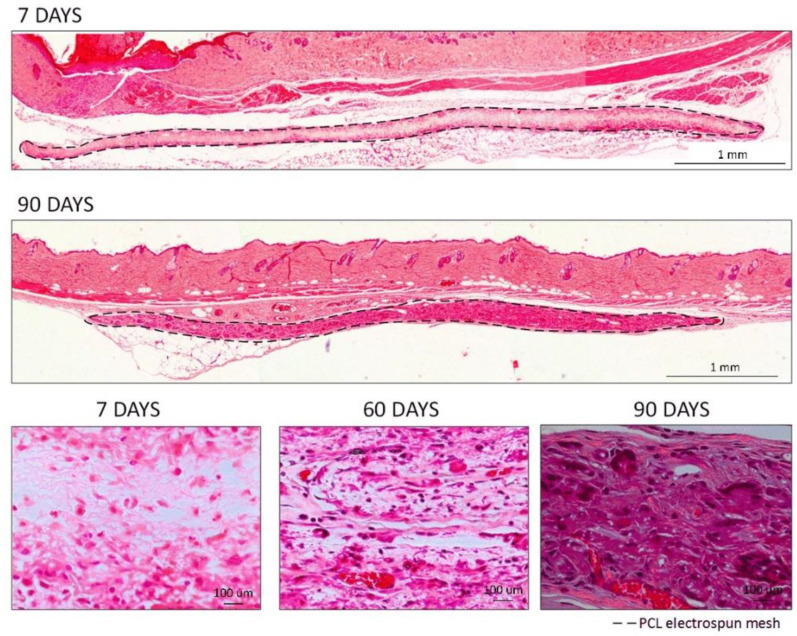
H&E staining for the comparison of the PCL electrospun meshes implanted during 7, 60, or 90 days.

When analyzing the images along time (Figure 6), it was clear that at the initial time-points, the host organism reacted to the electrospun meshes, but after 7 days, the inflammation processes were already slowing down. For the cells attached to the surface of the material and, at later time points (60 and 90 days), the analysis revealed that the cells were able to infiltrate the meshes and colonize the inner part. After 90 days, the cells that infiltrated the meshes were already producing extracellular matrix components (such as collagens, displayed in pink) that were probably occupying the place of the degraded PCL nanofibers. Hollow spaces could be observed as well as the presence of blood vessels penetrating the matrices within pores with larger diameters (with increased permanence time inside the host organism), thus demonstrating the biointegration of the PCL electrospun meshes.

## 4. Discussion

Surface and bulk erosion can describe how a degrading polymer erodes. In surface erosion, the polymer degrades from the exterior surface. The inside of the material does not degrade until all of the surrounding material around it has been degraded [37]. In bulk erosion, degradation occurs throughout the whole material equally (i.e., both the surface and the inside of the material degrade) [37]. Surface erosion and bulk erosion are not exclusive, since many materials undergo a combination of surface and bulk erosion [38]. In the electrospun PCL meshes, bulk erosion phenomena seem to have taken place, although further studies are needed.

The PCL degradation rate depends on its structural organization. However, in this field, there is great controversy around the evaluation of electrospun degradation in in vitro and/or in vivo conditions. Several works have explored the in vitro degradation of PCL electrospun meshes, be it either hydrolytic or enzymatic degradation. A study performed by Natu et al. evaluated the long-term hydrolytic degradation for PCL in fibers, sponges, films, and discs [39]. Their results demonstrated that the processing did not significantly affect the degradation rate during the advanced stage (18–36 months) [39]. In terms of weight loss, Castilla-Cortázar and co-workers demonstrated that PCL meshes, under enzymatic degradation with lipase and after 14 weeks, only lost 18% of weight [16]. According to Peng et al., the PCL electrospun fibers only lost 5% of weight in 18 days under enzymatic medium [16].

Some in vivo studies have been already carried out, evaluating the degradation of PCL-based electrospun meshes, although PCL was combined with other materials. In these studies, subcutaneous pockets were created to implant the meshes. According to Jiang and colleagues, after 1 month of implantation, histological and immunofluorescence evaluation showed continuous degradation of PCL/poly(trimethylene carbonate) (PTMC) and scaffolds induced a macrophage-mediated foreign body reaction [40]. In this study, degradation was correlated to the PTMC part since the average thickness of PCL remained constant during the experimental period [40]. Shi et al. evaluated the degradation of PCL/gelatin membranes with different ratios, over 24 weeks, and demonstrated that the incorporation of gelatin increased the biocompatibility and the biodegradation rate of the membranes [41]. Xue et al. also evaluated PCL/gelatin electrospun meshes in vivo over 6 months and demonstrated that after 12 weeks, the PCL samples degraded, although the samples with 70% of PCL with 30% of gelatin took only 1 week to degrade [42]. As far as we are aware, up to now, only Bölgen et al. have evaluated the degradation in vitro and in vivo of the PCL electrospun meshes alone by investigating the in vitro hydrolytic degradation over 6 months and in vivo degradation for 90 days [43]. In this study, the influence of the fiber diameter in the degradation rate was also evaluated, correlating the higher fiber surface/volume ratio with the higher degradation rate. The authors also clearly demonstrated that the in vivo degradation was faster than the hydrolytic one due to the presence of enzymes [43]. Accordingly, in the in vitro and in vivo studies described previously and regarding the non-conformable results, it is important to highlight that were used PCL with different molecular weights, different types of enzymes with different concentrations, and the degradation was monitored over different periods of times. Each parameter or all together directly influences the results compromising a correlation between the studies.

Despite the well-known PCL mechanical properties, it lacks in terms of biological activity, thus several studies have explored the combination of PCL with bioactive biomaterials, namely gelatin, collagen, cellulose, and hydroxyapatite [44,45,46,47]. In fact, the combination of PCL with other biomaterials also allows for an improvement in its degradation rate, as described by Hivechi et al.’s study [44], in which the degradation occurred 25% more rapidly when PCL/gelatin fibers were incorporated with cellulose nanocrystals. Another important strategy to tailor the PCL degradation rate is the development of advanced electrospun fibers/meshes, namely, core-shell fibers [48], bilayer meshes [49], and composite structures [50], allowing for a range of material ratios and surface area, making it possible to tailor the performance of the electrospun meshes such as the degradation kinetics. 

The present study compared the hydrolytic and enzymatic degradation with in vivo degradation, both during 90 days. In vitro enzymatic degradation clearly exhibited a faster degradation rate compared to the hydrolytic degradation. Moreover, the thermal results demonstrated the crystallinity of the PCL electrospun meshes varied as a function of degradation time. A crystallinity increase could be observed in both media, demonstrating that the crystallinity degree increased during hydrolysis. In fact, the amorphous regions have been reported to be prone to hydrolysis, leading to an increase in crystallinity during the degradation of semi-crystalline polyesters [16]. Additionally, the shorter chains formed during the degradation presented higher mobility, leading to a reorientation and increase in crystallinity [16]. After the degradation period, samples in the enzymatic medium reached 97.11% of weight loss, demonstrating the direct influence of enzymes in the degradation rate. The characterization of enzymatic degradation showed the erosion of the fiber surface throughout the degradation time. Consequently, the Young’s modulus increased and the elongation at break decreased due to the transition to a brittle structure. Regarding the in vivo studies, initially, a slight inflammatory reaction to the PCL electrospun meshes was observed due to the presence of some inflammatory cells but at day 7, the inflammation process was only residual. Posterior time-points (60 and 90 days) showed cellular infiltration throughout the meshes and, after 90 days, they were replaced by collagen, and the presence of blood vessels penetrating the PCL electrospun meshes could be observed. Furthermore, the histological images showed hollow spaces, demonstrating that some parts of the electrospun meshes were degraded, which constitutes a good indication of its usefulness for tissue regeneration applications. In vivo results demonstrated a clear biointegration of the electrospun meshes as well as the degradation of some parts of it.

In summary, degradation after 90 days was evident in both the in vitro and in vivo scenarios, demonstrating that PCL electrospun meshes are suitable for short-term TE applications, namely for skin tissue applications. However, due to the lack of the bioactivity of PCL electrospun meshes, its use as a single mesh is limited for wound healing application. Thus, it will be important to evaluate the degradation of electrospun meshes with PCL combined with other bioactive biomaterials and with advanced processing strategies such as core-shell or multilayer to establish the degradation kinetics for different conditions. Despite the results being promising, there is a gap between the in vitro and in vivo degradation rate that must be studied further, namely through the study of the in vivo degradation using a wound healing model and to evaluate in vitro more than one enzyme, as in most of the cases, the degradation rate is reduced due to the inhibitory action between them.

## 5. Conclusions

The in vitro degradation of electrospun meshes was monitored over 90 days in different media, namely hydrolytic or enzymatic, in order to evaluate its degradation rate for short-term applications such as skin tissue regeneration. After the degradation period, samples in the enzymatic medium reached 97.11% of weight loss, demonstrating the direct influence of enzymes in the degradation rate. The characterization of enzymatic degradation showed erosion of the fiber surface throughout the degradation time. Consequently, the Young’s modulus increased and the elongation at break decreased due to the transition to a brittle structure. The in vivo results demonstrated a clear biointegration of the electrospun meshes as well as degradation of some of its parts. In summary, degradation after 90 days was evident in both the in vitro and *i* scenarios, demonstrating that the PCL electrospun meshes are suitable for short-term applications, namely for tissue engineering. However, other ECM histological staining will be performed in the future.

## Figures and Tables

**Table 1:** The meshes’ dimensions and approximated surface area/volume ratio for the electrospun PCL meshes produced. The shape of the scaffold was assumed as a rectangular prism.

Structure	Length (mm)	Width (mm)	Thickness (mm)	Surface Area (SA) (mm^2^)	Volume (mm^3^)	SA/v (mm^−1^)
PCL electrospun mesh	40	10	0.17	97	6.8	14.3

**Table 2:** The characterization of the PCL electrospun meshes, depending on the degradation media.

Sample Type	Degradation Time (Days)	T_m_ (°C)	T_deg_ (°C)	M_n_ (kDa)	M_w_ (kDa)	PDI
PCL grain	NA	63.2 ± 0.4	386.8 ± 0.8	32.7 ± 9.7	45.5 ± 6.6	1.7
PCL mesh	NA	62.7 ± 0.7	376.9 ± 1.6	41.5 ± 4.2	57.8 ± 11.6	1.4
PBS	7	63.5 ± 1.0	379.1 ± 3.0	46.7 ± 3.4	60.0 ± 1.5	1.3
	14	64.3 ± 1.4	377.8 ± 1.9	47.5 ± 10.3	64.6 ± 15.8	1.4
	28	64.3 ± 0.4	376.9 ± 1.5	28.1 ± 6.2	50.1 ± 10.3	1.8
	42	64.6 ± 1.1	381.0 ± 1.3	39.0 ± 12.7	59.4 ± 18.2	1.5
	63	64.5 ± 0.8	380.2 ± 0.2	25.5 ± 6.5	42.6 ± 3.1	1.7
	77	64.9 ± 0.6	379.1 ± 0.4	35.3 ± 12.5	48.2 ± 9.9	1.4
	90	65.8 ± 0.1	381.0 ± 0.3	43.7 ± 3.7	59.7 ± 3.6	1.4
PBS/lipase	7	64.4 ± 0.5	380.7 ± 1.3	31.6 ± 11.8	48.0 ± 9.7	1.5
	14	63.9 ± 0.3	377.8 ± 3.6	37.0 ± 2.0	45.3 ± 0.8	1.2
	28	64.7 ± 0.3	379.0 ± 2.9	21.1 ± 5.8	33.0 ± 4.1	1.6
	42	65.7 ± 0.8	375.8 ± 2.2	33.0 ± 7.8	45.9 ± 1.1	1.4
	63	64.5 ± 0.8	370.0 ± 4.4	26.6 ± 9.0	51.0 ± 11.8	1.9
	77	65.4 ± 0.6	375.1 ± 2.9	38.5 ± 24.9	57.7 ± 2.9	1.5
	90	64.5 ± 0.9	374.3 ± 1.3	32.7 ± 9.7	45.5 ± 6.6	1.7

NA—Not applicable; M_n_—Number average molecular weight; M_w_—Weight average molecular weight; PDI—Polydispersity index; T_deg_—Degradation temperature; T_m_—Melting temperature.

## Data Availability

The raw data required to reproduce these findings cannot be shared at this time as the data also form part of an ongoing study.
